# Seaweed Extract (Stella Maris^®^) Activates Innate Immune Responses in *Arabidopsis thaliana* and Protects Host against Bacterial Pathogens

**DOI:** 10.3390/md16070221

**Published:** 2018-06-28

**Authors:** Jamie Cook, Janie Zhang, Jeff Norrie, Bachar Blal, Zhenyu Cheng

**Affiliations:** 1 Department of Microbiology and Immunology, Dalhousie University, Halifax, NS B3H 4R2, Canada; jamie.cook@dal.ca (J.C.); janiezhang@dal.ca (J.Z.); 2 Acadian Seaplants Limited, 30 Brown Avenue, Dartmouth, NS B3B 1X8, Canada; jpn@acadian.ca (J.N.); bblal@acadian.ca (B.B.)

**Keywords:** plant immunity, seaweed extract, plant protection, bacterial pathogen

## Abstract

Insects and pathogenic infections (bacteria, viruses and fungi) cause huge losses in agriculturally important crops yearly. Due to the rise in pesticide and antibiotic resistance, our crops and livestock are increasingly at risk. There is a rising demand for environmentally friendly solutions to prevent crop decreases. Components of *Ascophyllum nodosum* seaweed extracts were recently found to boost plant immunity. The stimulatory activities of the *A.* *nodosum* marine alga-derived extract (Stella Maris^®^) were investigated in a broad range of immune assays. Elevated hydrogen peroxide production measured in a chemiluminescence assay suggested that the extract elicited a strong burst of reactive oxygen species. *Arabidopsis* seedlings treated with Stella Maris^®^ activated the expression of *WRKY30*, *CYP71A12* and *PR-1* genes, the induction of which represent early, mid and late plant immune response, respectively. Finally, this study found that Stella Maris^®^ inhibited the growth of multiple bacterial pathogens, including an opportunistic human pathogen that has demonstrated pathogenicity in plants. In summary, the pre-treatment with the seaweed extract protected *Arabidopsis* against subsequent infection by these pathogens.

## 1. Introduction

As the world’s population is steadily increasing with no sign of stabilizing [1], a greater demand arises for a sustainable food supply [2]. With the impending threat of global warming and the rise of drug/pesticide resistant pests (bacteria, fungi, insects, etc.), our food sources are more vulnerable now than ever [3,4]. These problems affect many facets of food production including crops and livestock [4]. The use of synthetic pesticides on crop plants is declining due to reported side effects, which include air, soil and water contamination [5,6]. There is also pressure to reduce the use of antibiotics in the raising of farm animals, mainly due to increases in the number of antibiotic resistant human pathogens [7]. The use of genetically modified organisms (GMOs) is under debate due to the lack of knowledge about the safety of consuming these organisms [8,9]. Nevertheless, there are a number of GMO foods (mainly crop plants) approved for consumption within Canada and the United States [9]. There is also concern that GMO, when released into the environment may outcompete wild type organisms and transfer their transgenes by horizontal gene transfer [10]. Altogether, these factors underscore the need for agri- and aquacultural communities to develop alternative natural products to protect crops and animals.

Plant immune systems differ from their mammalian counterparts [11]. Mammals have both an innate and adaptive immune system, whereas plants have only an innate immune system [11]. Plant pathogens have many barriers to overcome before they can gain access to the host, which include a waxy cuticle, rigid cell walls, antimicrobial enzymes, and secondary metabolites [12]. Even if the pathogen gains entry into the host, innate immunity in plants is often triggered by the recognition of highly conserved microbe- or pathogen-associated molecular pattern (MAMP/PAMP) molecules, which include flagellin, peptidoglycan, lipopolysaccharides, cold shock proteins or chitin [12,13]. MAMP/PAMP triggered immunity leads to the activation of mitogen-activated protein kinases (MAPKs) and various hormone signaling pathways, which then start a cascade of defense responses that include alkalization of the growth medium, deposition of callose, production of reactive nitrogen/oxygen species (RNS/ROS), closure of the stomata, the production of antimicrobial compounds and various other secondary metabolites [12,13]. These defense responses allow plants to combat pathogenic infections [13]. 

Seaweeds contain many biological components including proteins, peptides, amino acids, fibers, lipids, pigments, phenols and polysaccharides, making them an attractive source for potential agricultural and health benefits [14,15]. Scientists have also tapped into seaweeds and seaweed extracts for both animal and human health benefits [16,17]. A study in rats has shown that feed supplementation with the red seaweed, *Chondrus crispus*, could have a prebiotic effect leading to an increase in the number of beneficial bacteria (*Bifodobacterium breve*) within the gut microbiome, eliciting a heightened immune response inside the host [18]. Similarly, in seaweed-consuming human populations (e.g., Japan) microbes are selected within the gut that produce carbohydrate active enzymes capable of breaking down the seaweed into pharmacologically active compounds, which have antiviral, anticancer or anti-inflammatory effects [19]. Other studies have shown that seaweeds contain many active compounds that demonstrate anti-cancer properties against human colon and breast cancers [20,21,22]. Research indicates that red seaweeds such as *Chondrus crispus* and *Sarcodiotheca gaudichaudii* can down-regulate virulence factors in *Salmonella enteritidis* and promote an immune response in *Caenorhabditis elegans* [23]. When supplemented in the feed of laying hens, these red seaweeds reduce the bacterial load of *Salmonella enteritidis* in the large intestine and excretions [24]. 

Seaweeds have been used for centuries by coastal farmers to fertilize their fields with the copious nutrients they contain [25]. It is not surprising that seaweed products also contain plant growth-promoting (PGP) compounds [26]. This PGP activity of seaweed products can be direct (e.g., fertilizer/nutrients for plants) [26], or indirect (e.g., by providing nutrients for beneficial PGP rhizobacteria (PGPR), which in turn promote plant growth) [27]. For example, inoculating wheat with both seaweed extracts and PGPR increased the PGPR’s halotolerance, which led to greater plant mass than inoculating with either treatment alone [28]. Some recent studies have demonstrated the ability of seaweed extracts to promote immune responses in plants and that specific components of such extracts can promote various facets of plant immunity [29]. Ulvans and oligo-ulvans extracted from *Ulva fasciata* induced the expression of enzymes within the phenylpropanoid pathway in *Medicago truncatula* [30]. The oligo-alginate Polymannuronic acid produced by acid hydrolysis induced phenylalanine ammonia lyase (PAL) activity in wheat leaves maximally at 24 h [31]. Fucans and oligo-fucans prepared by enzyme digestion of fucans induced the release of hydrogen peroxide in tobacco cells maximally at 6 min post-treatment [32]. Laminarin extracted from the brown seaweed *Laminaria digitata* protected tobacco plants against *Erwinia carotovora* by reducing the diameter of necrotic lesions [33]. Carrageenans and oligo-carrageenans induced the expression of a chitinase, with antifungal properties, and a type II proteinase inhibitor in tobacco leaves [34]. These studies have all looked at individual components of seaweeds, and little is known about the molecular mechanisms by which these seaweed components promote plant health. 

In the present study, the ability of an *Ascophyllum nodosum* extract, Stella Maris^®^ (Acadian Seaplants), to elicit an immune response was evaluated in the model plant *Arabidopsis thaliana.* A wide range of immune assays were used to examine immuno-modulatory capabilities of the *Ascophyllum* extract*,* including histochemical staining of defense-reporter plant tissues, hydrogen peroxide production, defense-related gene measurement using reverse transcription and quantitative polymerase chain reaction (RT-qPCR). Importantly, it was found that Stella Maris^®^ can directly inhibit the growth of a diverse range of bacterial pathogens. Finally, it was shown that the *Ascophyllum* extract protected *A. thaliana* against the infection caused by these pathogens.

## 2. Results

### 2.1. Stella Maris^®^ Activates Innate Immune Responses in Arabidopsis thaliana

**Figure 1:**
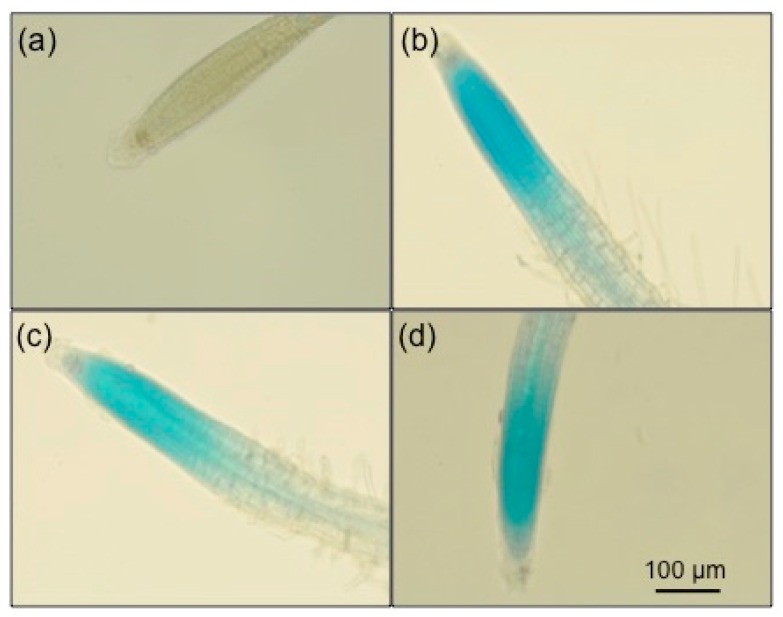
GUS Histochemical assay showing elicitation of the β-glucuronidase (GUS) reporter gene, put under the control of the pathogen-inducible CYP71A12 promoter (CYP71A12pro:GUS) in *Arabidopsis thaliana* (CYP71A12) following a 6-h treatment with various concentrations of Stella Maris^®^. Images were captured using the 20× objective lens. (**a**) Control seedling that was untreated. (**b**–**d**) were treated with 0.05%, 0.5% and 5%, respectively for 6 h prior to staining with GUS substrate solution. The experiments were repeated three times with the same result.

To determine whether *Arabidopsis thaliana* elicited an immune response towards Stella Maris^®^, the β-glucuronidase (GUS) histochemical assay was used. This assay was developed to investigate whether *Arabidopsis* roots respond to MAMPs/PAMPs, and if so, which cell types respond to such markers [35]. The GUS histochemical assay also allowed us to determine the location of the immune response [35]. In this case, the GUS reporter gene was put under the control of the pathogen-inducible *CYP71A12* promoter (CYP71A12pro:GUS). *CYP71A12* is inducible in the presence of immune elicitors such as bacterial flagellin, elongation factor Tu, and a bacterially secreted protease (protease IV from *P. aeruginosa*) [36]. All concentrations (0.05%, 0.5% and 5%) of Stella Maris^®^ elicited strong immune responses in the transgenic CYP71A12pro:GUS *Arabidopsis* plants (Figure 1b–d). There was no induction of the promoter in the stained negative control seedlings (Figure 1a). 

**Figure 2:**
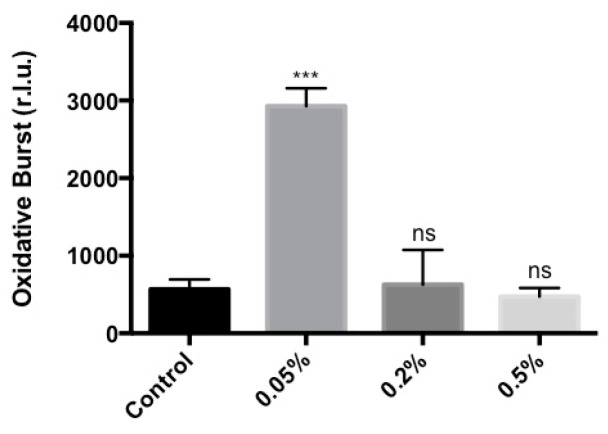
Chemiluminescence assay measuring elicitation of an oxidative burst after 9 min of incubation of various concentrations (0.05%, 0.2% and 0.5%) of Stella Maris^®^ and ROS reaction mixture; r.l.u: relative luminescence units. Error bars indicate standard deviation generated from 3 biological replicates. *** *p* ≤ 0.001; ns: Not significant at the 0.05 probability level. The experiments were repeated three times with the same result.

It was then determined whether the treatment of *Arabidopsis* with *Ascophyllum* extract activated other recognized plant defense responses. For example, reactive oxygen species (ROS) production in *Arabidopsis* is a response to invading pathogens. Specifically, hydrogen peroxide production can inhibit attacks from bacteria, viruses and fungi [37]. In addition, hydrogen peroxide production caused by pathogenic infections activates *WRKY* transcription factors, which in turn induce salicylic acid (SA) and jasmonic acid (JA) innate immunity pathways in *Arabidopsis* [38]*.* Therefore, ROS production was used as an indicator of a relatively upstream immune signaling event in *Arabidopsis* plants, and measured using a chemiluminescence assay. Maximum ROS production in *Arabidopsis* seedlings occurred after 9 min of incubation with Stella Maris^®^ and the ROS reaction mixture. A 0.05% concentration of the *Ascophyllum* extract produced the highest amount of ROS, which was approximately 4.2 fold greater than the background level in control plants (Figure 2 and Figure S1). In contrast, *Arabidopsis* seedlings treated with higher concentrations of *Ascophyllum* extract did not produce an obvious oxidative burst when compared to the control. This may be due to the nature of the assay and/or the colour of the extract (Figure S2). The ROS assay is a chemiluminescence-based assay that relies on the production of light to measure the amount of hydrogen peroxide produced. Therefore, since the elicitor is a dark solution, the colour may interfere with the detection of light (Figure S2). 

**Figure 3:**
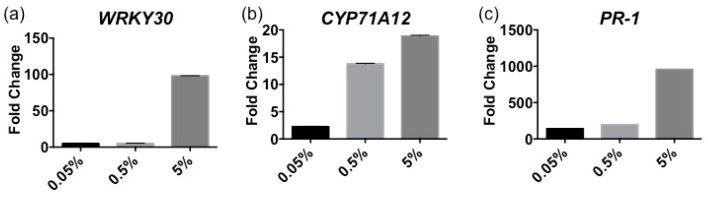
Induction of defence-related gene expression by various concentrations of Stella Maris^®^ (0.05%, 0.5% and 5%) in wild-type Col-0 measured by RT-qPCR. (**a**) Relative gene expression of *WRKY30* at 1 h post-treatment. (**b**) Relative gene expression of *CYP71A12* at 6 h post-treatment. (**c**) Relative gene expression of *PR-1* at 24 h post-treatment. Error bars indicate standard deviation generated from 3 technical replicates of 10 *Arabidopsis thaliana* seedlings. Each experiment includes 10 individual plants under each condition. The experiments were repeated three times with the similar results.

Plant immune response is tightly controlled by a cascade of signaling events. Previous studies have characterized how the immune signals are orchestrated by diverse immune regulators [12,13]. The transcriptomic responses in plants treated by MAMPs/PAMPs have been well characterized using RNA sequencing, coupled with RT-qPCR [36]. The mechanism by which *Ascophyllum* extract activates defense-related effectors was examined during various stages of the immune response using RT-qPCR to measure the relative gene expression that correlates to early, mid and late immune responses. The expression of *WRKY30, CYP71A12* and *PR-1* were measured at 1-, 6- and 24-h post treatment with Stella Maris^®^, respectively. The RT-qPCR data indicate the relative gene expression of *Arabidopsis* seedlings treated with three concentrations of *Ascophyllum* extract compared to the buffer-treated controls (Figure 3a–c). *Arabidopsis* seedlings treated with 5% extract showed the strongest immune activation for all genes measured (Figure 3a–c). At 1 h there was a 98-fold increase in expression of *WRKY30* in plants treated with 5% Stella Maris^®^ (Figure 3a). Similarly, a 19-fold increase in the expression of *CYP71A12* occurred at 6 h in plants treated with 5% of the extract (Figure 3b). Finally, at 24 h post treatment with 5% Stella Maris^®^, a 1000-fold increase in expression of *PR-1* was observed (Figure 3c). 

### 2.2. Stella Maris^®^ Inhibits the Growth of Multiple Bacteria

**Figure 4:**
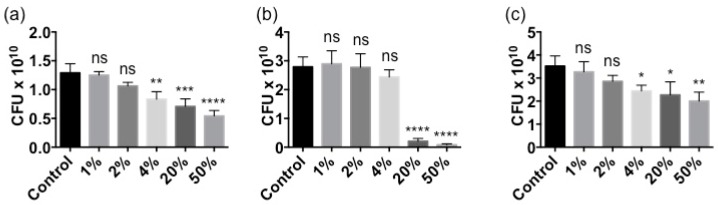
Inhibition of bacterial growth at various concentrations (1%, 2%, 4%, 20% and 50%) of Stella Maris^®^. (**a**) *P. syringae* DC3000’s CFU measured after incubation with various concentrations of Stella Maris^®^ overnight at 30 °C. (**b**) *P. aeruginosa* PA14’s CFU measured after incubation with various concentrations of Stella Maris^®^ overnight at 37 °C. (**c**) *X. campestris* BP109’s CFU measured after incubation with various concentrations of Stella Maris^®^ overnight at 30 °C. Error bars indicate standard deviation generated from 3 biological replicates; CFU: colony forming units; **** *p* ≤ 0.0001; *** *p* ≤ 0.001; ** *p* ≤ 0.01; * *p* ≤ 0.05; ns: Not significant at the 0.05 probability level.

*Ascophyllum* extract-triggered immune response was examined to identify whether it could help protect against pathogenic infections. Before evaluating whether the plant defense response boosted by the extract could prime their immunity to fend off pathogens, the ability of the *Ascophyllum* extract to directly inhibit the growth of bacterial pathogens was investigated. The direct anti-microbial activity of Stella Maris^®^ has not been documented previously. The number of colony forming units (CFU) was measured to assess whether Stella Maris^®^ inhibited the growth of the well-characterized plant pathogen *P. syringae* DC3000. In the presence of 4%, 20% and 50% concentrations of Stella Maris^®^ in LB broth, the overnight growth of *P. syringae* was reduced 35%, 45% and 58%, respectively, compared to the control (LB alone) (Figure 4a). There were no significant differences in growth between the control and the 1% and 2% concentrations of Stella Maris^®^ in LB broth (Figure 4a). The extract also inhibited the growth of a broad-host-range opportunistic pathogen *Pseudomonas aeruginosa*. However, much higher concentrations, 20% and 50%, of Stella Maris^®^ were needed to inhibit *P. aeruginosa* by 93% and 97%, respectively (Figure 4b). There was no significant difference in growth between the control and 1%, 2% and 4% concentrations of Stella Maris^®^ in LB broth (Figure 4b). Finally, the inhibition noted in *Pseudomonas* was tested for applicability to another genus of plant pathogens. Incubation of Stella Maris^®^ at concentrations of 4%, 20% and 50% in NYG broth inhibited the growth of *Xanthomonas campestris* by 31%, 36% and 43%, respectively (Figure 4c). No significant difference was observed in growth between the control and 1% and 2% concentrations of Stella Maris^®^ in NYG broth (Figure 4b). 

### 2.3. Stella Maris^®^ Protects Arabidopsis thaliana against Bacterial Pathogens

**Figure 5:**
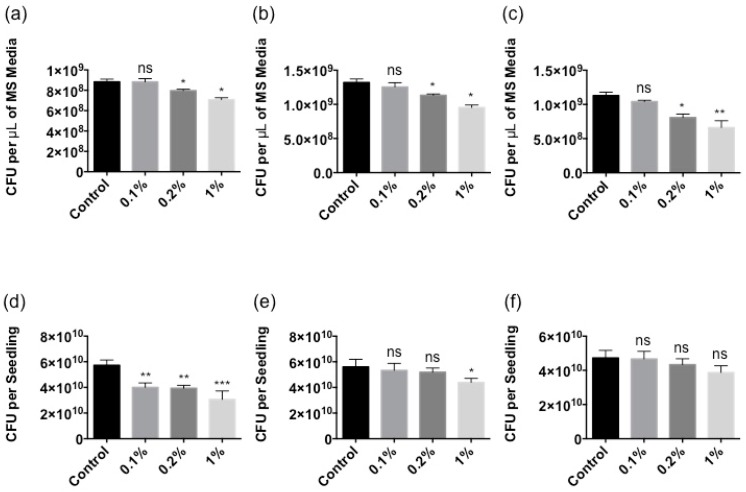
Treatment with 0.1%, 0.2% and 1% of Stella Maris^®^ showing the reduction of bacterial burden in plant growth media and within *Arabidopsis* tissues. (**a**) *P. syringae* DC3000 CFU measured in the MS media after 24 h of treatment. (**b**) *P. aeruginosa* PA14 CFU measured in the MS media after 24 h of treatment. (**c**) *X. campestris* BP109 CFU measured in the MS media after 24 h of treatment. (**d**) *P. syringae* DC3000 CFU measured in the plant tissue at 28 h. (**e**) *P. aeruginosa* PA14 CFU measured in the plant tissue at 28 h. (**f**) *X. campestris* BP109 CFU measured in the plant tissue at 28 h; CFU: colony forming units; *** *p* ≤ 0.001; ** *p* ≤ 0.01; * *p* ≤ 0.05; ns: Not significant at the 0.05 probability level.

Based on the data above, the protection assay was performed as follows. At higher concentrations (>4% as shown in Figure 4), Stella Maris^®^ demonstrated a direct inhibitory effect on bacterial growth. Therefore, concentrations that didn’t show a detrimental outcome on bacterial growth were chosen. However, they were still effective at eliciting a robust immune response and were tested in a pretreatment experiment. To avoid potential complications from the presence of the *Ascophyllum* extract on bacterial growth, it was removed from the plant growth media after boosting plant immune response and treated plants were washed several times with plant growth medium before inoculating with the bacterial pathogens. The CFU’s of *P. syringae*, *P. aeruginosa* and *X. campestris* were measured to determine whether pretreatment with Stella Maris^®^ provided protection. With regard to bacterial load in the MS media (exterior to the plant), 1% Stella Maris^®^ provided the greatest level of induction of protection against all three bacterial pathogens at all times measured (Figure 5a–c and Figure S3a–c). Furthermore, it reduced bacterial load at 24 h by 20%, 28% and 41% in plants infected by *P. syringae*, *P. aeruginosa* and *X. campestris*, respectively (Figure 5a–c). Stella Maris^®^ at a concentration of 0.2% also induced protection against the bacterial pathogens, however to a lesser extent (10%, 14% and 28%, respectively) (Figure 5a–c). In all cases, no induced protection was observed against pathogens treated with 0.1% of Stella Maris^®^ (Figure 5a–c). Pretreatment with 0.1%, 0.2% and 1% concentrations of Stella Maris^®^ also reduced *P. syringae* bacterial load within *Arabidopsis* tissue by 30%, 31% and 47%, respectively (Figure 5d). *Arabidopsis* pretreated with Stella Maris^®^ at a concentrations of 1% reduced *P. aeruginosa* bacterial burden within the plant tissues by 22% relative to the control (Figure 5e). However, no significant protection was afforded against *X. campestris* bacterial burden within *Arabidopsis* tissues (Figure 5f). 

## 3. Discussion

With the increase in pesticide resistance among many plant pathogens, and harmful environmental effects found with these pesticides, there is a need for safe biologically active compounds that can boost plant immune response and inhibit the growth of pathogenic fungi and bacteria [5]. Stella Maris^®^ is a commercially available *Ascophyllum* extract with known PGP capabilities [39]. However, there is little current evidence that this extract can boost plant immunity. Seaweed extracts have been fractioned into biologically active compounds such as ulvans and oligo-ulvans, oligo-alginate, fucans and oligo-fucans, laminarin and carrageenans and oligo-carrageenans [29]. However, studies have not looked at seaweed extracts as a whole, and in many cases the individual biological components of the extracts only activated one plant immune response pathway [30,31,32,33,34]. By treating plants with complete seaweed extract, it is hypothesized to be more beneficial to the plant by activating several pathways within the innate immune response.

Stella Maris^®^ immune modulating abilities were assessed in *A. thaliana*, and our results showed that the extract activated a strong innate immune response in *Arabidopsis* as evidenced by well-characterized high-throughput assays. The ROS chemiluminescence assay showed that the *Ascophyllum* extract activates a rapid (9 min post-treatment) immune response in the form of hydrogen peroxide production (Figure 2). Plants treated with lower concentrations (0.05%) of this extract produced more ROS compared to higher concentrations (0.2% and 0.5%), a result most likely due to the fact that the *Ascophyllum* extract is naturally dark brown. Therefore, higher concentrations are less transparent making it more difficult for light to be detected by the plate reader. It is suggested that if all samples had similar transparency, a ROS response from the higher concentrations of the *Ascophyllum* extract would have been detected. Nonetheless, the extract was shown to be capable of eliciting a strong immune response in the form of hydrogen peroxide production. *Arabidopsis* seedlings treated with the *Ascophyllum* extract (0.05%) produced 0.8-fold more hydrogen peroxide compared to plants treated with 100 nM of flg22 (Figure S1). This is highly significant given that flg22 is a synthetic flagellar peptide, well known to produce a very strong immune response in *Arabidopsis* [40]. 

Subsequently, it was found that the *Ascophyllum* extract activated the expression of a transcription factor, *WRKY30*, which responds to hydrogen peroxide production and is involved in the expression of genes within the SA and JA immunity signaling pathways [41]. SA and JA are important plant defense hormones that have been shown to deter pathogens [42]. SA is more effective against biotrophic (living plant tissues) pathogens, whereas JA is better deterrent for necrotrophic (cell-death provoking) pathogens [42]. 

The *Ascophyllum* extract also elicited a strong immune response in *Arabidopsis* seedlings 6 h post-treatment as indicated by the expression of *CYP71A12*. It was reported that *CYP71A12* expression is maximally induced at the 6-h time point [36]. Using CYP71A12pro:GUS reporter transgenic plants, the activation of an immune response by *Ascophyllum* extract was found in the elongation zone of the root tip. Further, an increase in the expression of *CYP71A12* in the seedlings treated with the *Ascophyllum* extract was observed relative to the untreated control using RT-qPCR. *CYP71A12* is a cytochrome P450 family polypeptide responsible for the production of camalexin, a phytoalexin with antimicrobial functions that damage cell walls and disrupt metabolism in attacking pathogens [43]. For this reason, phytoalexins are known to accumulate in areas of infection [43]. A similar immune activation pattern is seen when the immune response is elicited with bacterial flagellin in *Arabidopsis* [35,36]. 

Finally, Stella Maris^®^ was examined for its ability to sustain an immune response. The expression of PR-1, a defense gene regulated by SA production involved in systemic acquired resistance to attacking pathogens, was measured [44]. The PR-1 gene expression was experimentally determined to be maximally induced by bacterial flagellin at the 24-h time point [45]. Consistently, the 24-h time point showed a strong activation of *PR-1* by the *Ascophyllum* extract in *Arabidopsis* seedlings, therefore we chose to examine the protective capabilities of Stella Maris^®^ in *Arabidopsis* when challenged with diverse bacterial pathogens. A decrease in the number of bacteria found free-living in the MS media was observed as well as a decrease in bacteria found within the seedlings were pre-treated with the *Ascophyllum* extract for 24 h. The decrease in bacteria free-living within the MS media is likely due to the production of hydrogen peroxide and other antimicrobial compounds by the *Arabidopsis* seedlings, whereas the decrease in bacteria found within the plant tissue may be due to strengthening of the plant cell wall [45]. It was important for the plant protection assay that the Stella Maris^®^ be washed away prior to bacterial infection because the extract also has a noticeable inhibitory effect on bacterial growth. 

## 4. Materials and Methods

### 4.1. Plant Growth

*A. thaliana* seeds were surface sterilized using equal parts of bleach, sterile water and 70% ethanol for 5 min, then washed three times using sterile water and stored at 4 °C for two days prior to planting. Seedlings were grown in MS liquid media (Murashige and Skoog basal medium with vitamins from Phytotechnology Laboratories supplemented with 0.5% sucrose and 0.5 g L^−1^ MES hydrate buffered to pH 5.7 using 1N potassium hydroxide, Lenexa, KA, USA) in either 24-well plates (BD Falcon, Bedford, MA, USA) (ten seeds and 0.5 mL medium per well) for GUS expression, RT-qPCR and protection assays, or 96-well plates (Greiner Bio-One, Kremsmünster, Austria) (one seed and 0.2 mL medium per well) for oxidative burst measurements. In both cases plates were sealed with Micropore tape and placed on a grid-shelve over water on a growth light stand (Hydrofarms, Fairless Hills, PA, USA) for 10 days at 22 °C, under 16 h of daylight (100 μE m^−2^ s^−1^). The media in the 24-well plates was exchanged for fresh media on day 8, while the media in the 96-well plate was exchanged for sterile water on day 9.

### 4.2. Elicitor Treatments

Optimal concentrations of Stella Maris^®^, an extract of the brown seaweed *Ascophyllum nodosum*, were experimentally determined to be 0.05%, 0.5% and 5% in sterile water for GUS expression, ROS measurement and RT-qPCR, and 0.1%, 0.2% and 1% for the protection assay. Ten-day-old seedlings were treated with the different elicitors for the following times: 6 h for GUS assay in reporter line *CYP71A12pro:GUS*: 1 h, 6 h and 24 h for RT-qPCR analysis of selected genes. Nine-day-old seedlings were treated with the different elicitors for 24 h prior to the protection assay.

### 4.3. GUS Histochemical Assay

After plants were treated with either 0.05%, 0.5% or 5% concentrations of Stella Maris^®^ for 6 h, the plants were then washed with 50 mM sodium phosphate (pH 7), and 0.5 mL of GUS substrate solution (50 mM sodium phosphate, pH 7, 10 mM EDTA, 0.5 mM K_4_[Fe(CN)_6_], 0.5 mM K_3_[Fe(CN)_6_], 0.5 mM X-Gluc (5-bromo-4-chloro-3-indolyl-beta-d-glucuronic acid, cyclohexylammonium salt), and 0.1% *v*/*v* Triton X-100) was added to each well. The plants were then incubated at 37 °C for 4 h. Plant tissues were fixed with a 3:1 ethanol:acetic acid solution at 4 °C overnight and then placed in 95% ethanol. Plant tissues were cleared in lactic acid and examined using a Nikon DIAPHOT-TMD inverted microscope (Tokyo, Japan). 

### 4.4. Oxidative Burst Measurement

H_2_O_2_ production in plants was detected using a luminol-horse radish peroxidase (HRP)-based chemiluminescence assay. A 10 mg mL^−1^ of 500× HRP (Sigma-Aldrich, St. Louis, MO, USA) stock solution was prepared by dissolving 10 mg HRP in sterile water. A 20 mg mL^−1^ of 500× luminol (Sigma-Aldrich, St. Louis, MO,USA) stock solution was prepared by dissolving 20 mg luminol in 100 mM KOH. For each elicitor concentration of Stella Maris^®^, a master reaction mixture was prepared by diluting the elicitor in HRP, and luminol stock solutions in sterile water. The 96 well plates containing the seedlings were kept in the dark for 1 h prior to the treatment with each respective elicitor. The following procedures were completed in a dark room. Water was removed from each well within the 96 well plate following the 1 h pretreatment in the dark, and 200 μL of elicitor master reaction mixture was added to each well. The plate was then placed in a 96 well plate reader VICTOR™X5 (PerkinElmer, Waltham, MA, USA), and read for 30 cycles. The kinetics of H_2_O_2_ production was determined by plotting the average of chemiluminescence counts from all the seedlings placed under the same elicitor treatment over the measurement period. 

### 4.5. RNA Isolation and RT-qPCR Analysis

Total plant RNA was isolated according to the manufacturers’ instructions using an RNeasy Plant Mini Kit (Qiagen, Hilden, Germany). DNA was removed from all of the RNA samples using the DNA-free kit (Invitrogen, Carlsbad, CA, USA), and reverse transcription reactions were performed using an iScript cDNA synthesis kit (Bio-Rad, Hercules, CA, USA). Complementary DNA (cDNA) concentrations were measured using a Nano-drop instrument (Thermo Scientific, Waltham, MA, USA). RT-qPCR reactions were performed using CFX96 real-time PCR machine (Bio-Rad, Hercules, CA, USA) using SsoAdvanced™ Universal SYBR^®^ Green Supermix (Bio-Rad, Hercules, CA, USA). PCR reactions were performed as follows: 95 °C for 3 min followed by 40 cycles of 95 °C for 10 s and 55 °C for 30 s. Fold change was calculated relative to plants treated with sterile water. Gene induction values represent the mean ± s.d., *n* = 3 with each containing ten seedlings. Gene expression values were normalized to the eukaryotic translational initiation factor 4A1 (*EIF4A1*). The following primers were used: *EIF4A1* (At3g13920), 5′-GCAGTCTCTTCGTGCTGACA-3′ and 5′-TGTCATAGATCTGGTCCTTGAA-3′; *CYP71A12* (At2g30750), 5′-GATTATCACCTCGGTTCCT-3′ and 5′-CCACTAATACTTCCCAGATTA-3′; *WRKY30* (At5g24110) 5′-GCAGCTTGAGAGAGCAAGAATG-3′ and 5′-AGCCAAATTTCCAAGAGGAT-3′; *PR1* (At2g14610) 5′-CCTTCTCGGTGATCCATTCT-3′ and 5′-GTGCAATGGAGTTTGTGGTC-3′. *WRKY30*, *CYP71A12* and *PR1* gene inductions were measured after 1 h, 6 h and 24 h, respectively. 

### 4.6. Bacterial Growth

*Pseudomonas syringae* DC3000 and *Xanthomonas campestris* BP109 were grown overnight for 16 h at 30 °C. *Pseudomonas aeruginosa* PA14 was grown overnight for 16 h at 37 °C. Overnight cultures were centrifuged at 5000× *g* for 10 min, washed twice with 10 mM MgSO_4_. The pathogens were resuspended separately in MS liquid media for the protection assay. *P. syringae* DC3000 and *P. aeruginosa* PA14 were resuspended in LB broth for growth inhibition assays. *X. campestris* was resuspended in NYG (5 g of peptone, 3 g of yeast extract, and 20 g of glycerol per litre) broth for the growth inhibition assay. Bacterial optical densities were adjusted to 0.0002 and 0.01 for the protection and growth inhibition assays, respectively.

### 4.7. Growth Inhibition Assay

Bacterial cultures were grown overnight for 16 h in various concentrations of Stella Maris^®^ (1%, 2%, 4%, 20% and 50%) in either LB broth for *P. syringae* DC3000 and *P. aeruginosa* PA14 or NYG broth for *X. campestris* BP109, then serially diluted from 10^0^–10^−7^. The plates were grown at room temperature (22 °C) overnight, and CFU were counted the following day.

### 4.8. Protection Assay

The elicitors were removed on day 10 of the assay, by washing the plants three times using sterile water, before adding either *P. syringae* DC3000*, P. aeruginosa* PA14 or *X. campestris* BP109 to each well. Liquid media samples were taken from each well at 16 h, 20 h, and 24 h. The liquid media were serially diluted from 10^0^–10^−7^, and plated on LB plates. The plants within each well were collected at 28 h post-infection, and their tissues were homogenized and plated on LB or NYG plates. The plates were incubated at room temperature (22 °C) overnight, and CFU were counted the following day.

## 5. Conclusions

The *Ascophyllum nodosum* extract, Stella Maris^®^, was found to modulate early-, mid- and late-immune responses in the model organism *Arabidopsis*. The extract not only directly inhibits the growth of all three bacterial pathogens tested, including an opportunistic human pathogen, it also activates a strong immune response in plants that had a protective effect against these bacterial pathogens. This study suggests that seaweed extracts are effective biofertilizers due to the activation of the innate immune response in plants. Moving forward, seaweed extracts are an environmentally friendly option to enhance crop yield. 

## Supplementary Materials

The following are available online at http://www.mdpi.com/1660-3397/16/7/221/s1, Figure S1: Chemiluminesence assay showing elicitation of an oxidative burst by Stella Maris^®^, r.l.u.: relative luminescence units. Figure S2: Various concentrations of Stella Maris^®^. Figure S3: Reduction of bacterial burden in plant growth media at 16 h and 20 h.

## References

[1] Gerland P., Raftery A.E., Ševěíková H., Li N., Gu D., Spoorenberg T., Alkema L., Fosdick B.K., Chunn J., Lalic N. (2014). World population stabilization unlikely this century. Science.

[2] Balatsky A.V., Balatsky G.I., Borysov S.S. (2015). Resource demand growth and sustainability due to increased world consumption. Sustainability.

[3] Wheeler T., Von B.J. (2013). Climate change impacts on global food security. Science.

[4] Orzech K.M., Nichter M. (2008). From resilience to resistance: Political ecological lessons from antibiotic and pesticide resistance. Annu. Rev. Anthropol..

[5] Gavrilescu M., Demnerová K., Aamand J., Agathos S., Fava F. (2015). Emerging pollutants in the environment: Present and future challenges in biomonitoring, ecological risks and bioremediation. New Biotechnol..

[6] Pimentel D., Burgess M. (2014). Environmental and economic costs of the application of pesticides primarily in the United States. Integr. Pest Manag..

[7] Woolhouse M., Ward M., van Bunnik B., Farrar J. (2015). Antimicrobial resistance in humans, livestock and the wider environment. Philos. Trans. R. Soc. B Biol. Sci..

[8] Blancke S., Van Breusegem F., De Jaeger G., Braeckman J., Van Montagu M. (2015). Fatal attraction: The intuitive appeal of GMO opposition. Trends Plant Sci..

[9] Bawa A.S., Anilakumar K.R. (2013). Genetically modified foods: Safety, risks and public concerns—A review. J. Food Sci. Technol..

[10] Keese P. (2008). Review article risks from GMOs due to horizontal gene transfer. Environ. Biosaf. Res..

[11] Ronald P.C., Beutler B. (2010). Plant and animal sensors of conserved microbial signatures. Science.

[12] Muthamilarasan M., Prasad M. (2013). Plant innate immunity: An updated insight into defense mechanism. J. Biosci..

[13] Jones J.D.G., Dangl J.L. (2006). The plant immune system. Nature.

[14] Liu J., Hafting J., Critchley A.T., Banskota A.H., Prithiviraj B. (2013). Components of the cultivated red seaweed *Chondrus crispus* enhance the immune response of *Caenorhabditis elegans* to *Pseudomonas aeruginosa* through the pmk-1, daf-2/daf-16, and skn-1 pathways. Appl. Environ. Microbiol..

[15] Pérez M.J., Falqué E., Domínguez H. (2016). Antimicrobial action of compounds from marine seaweed. Mar. Drugs.

[16] O’Sullivan L., Murphy B., McLoughlin P., Duggan P., Lawlor P.G., Hughes H., Gardiner G.E. (2010). Prebiotics from marine macroalgae for human and animal health applications. Mar. Drugs.

[17] Wells M.L., Potin P., Craigie J.S., Raven J.A., Merchant S.S., Helliwell K.E., Smith A.G., Camire M.E., Brawley S.H. (2017). Algae as nutritional and functional food sources: Revisiting our understanding. J. Appl. Phycol..

[18] Liu J., Kandasamy S., Zhang J., Kirby C.W., Karakach T., Hafting J., Critchley A.T., Evans F., Prithiviraj B. (2015). Prebiotic effects of diet supplemented with the cultivated red seaweed *Chondrus crispus* or with fructo-oligo-saccharide on host immunity, colonic microbiota and gut microbial metabolites. BMC Complement. Altern. Med..

[19] Hehemann J.-H., Kelly A.G., Pudlo N.A., Martens E.C., Boraston A.B. (2012). Bacteria of the human gut microbiome catabolize red seaweed glycans with carbohydrate-active enzyme updates from extrinsic microbes. Proc. Natl. Acad. Sci. USA.

[20] Moussavou G., Kwak D.H., Obiang-Obonou B.W., Maranguy C.A.O., Dinzouna-Boutamba S.D., Lee D.H., Pissibanganga O.G.M., Ko K., Seo J.I., Choo Y.K. (2014). Anticancer effects of different seaweeds on human colon and breast cancers. Mar. Drugs.

[21] Ulloa A., Gonzales A.L., Zhong M., Kim Y., Cantlon J., Ku C., Earley S., Sanborn B.M., Mcmaster M.L., Kristinsson S.Y. (2009). Growth-inhibitory effects of a mineralized extract from the red marine algae, *Lithothamnion calcareum*, on Ca^2+^-sensitive and Ca^2+^-resistant human colon carcinoma cells. Cancer Lett..

[22] Kim E.J., Park S.Y., Lee J.-Y., Park J.H.Y. (2010). Fucoidan present in brown algae induces apoptosis of human colon cancer cells. BMC Gastroenterol..

[23] Kulshreshtha G., Borza T., Rathgeber B., Stratton G.S., Thomas N.A., Critchley A., Hafting J., Prithiviraj B. (2016). Red seaweeds *Sarcodiotheca gaudichaudii* and *Chondrus crispus* down regulate virulence factors of *Salmonella enteritidis* and induce immune responses in *Caenorhabditis elegans*. Front. Microbiol..

[24] Kulshreshtha G., Rathgeber B., MacIsaac J., Boulianne M., Brigitte L., Stratton G., Thomas N.A., Critchley A.T., Hafting J., Prithiviraj B. (2017). Feed supplementation with red seaweeds, *Chondrus crispus* and *Sarcodiotheca gaudichaudii*, reduce *Salmonella enteritidis* in laying hens. Front. Microbiol..

[25] A Guide to the Seaweed Industry. ftp://ftp.fao.org/docrep/fao/006/y4765e/y4765e00.pdf.

[26] Briceño-Domínguez D., Hernández-Carmona G., Moyo M., Stirk W., van Staden J. (2014). Plant growth promoting activity of seaweed liquid extracts produced from *Macrocystis pyrifera* under different pH and temperature conditions. J. Appl. Phycol..

[27] Arioli T., Mattner S.W., Winberg P.C. (2015). Applications of seaweed extracts in Australian agriculture: Past, present and future. J. Appl. Phycol..

[28] Rai A., Cherif A., Cruz C., Nabti E. (2017). Extracts from seaweeds and *Opuntia ficus-indica* Cladodes enhance diazotrophic-PGPR halotolerance, their enzymatic potential, and their impact on wheat germination under salt stress. Pedosphere.

[29] Vera J., Castro J., Gonzalez A., Moenne A. (2011). Seaweed polysaccharides and derived oligosaccharides stimulate defense responses and protection against pathogens in plants. Mar. Drugs.

[30] Cluzet S., Torregrosa C., Jacquet C., Lafitte C., Fournier J., Mercier L., Salamagne S., Briand X., Esquerré-Tugayé M.T., Dumas B. (2004). Gene expression profiling and protection of *Medicago truncatula* against a fungal infection in response to an elicitor from green algae *Ulva* spp.. Plant Cell Environ..

[31] Chandía N.P., Matsuhiro B., Mejías E., Moenne A. (2004). Alginic acids in *Lessonia vadosa*: Partial hydrolysis and elicitor properties of the polymannuronic acid fraction. J. Appl. Phycol..

[32] Klarzynski O., Descamps V., Plesse B., Yvin J.-C., Kloareg B., Fritig B. (2003). Sulfated fucan oligosaccharides elicit defense responses in tobacco and local and systemic resistance against tobacco mosaic virus. Mol. Plant Microbe Interact..

[33] Klarzynski O., Plesse B., Joubert J.-M., Yvin J.-C., Kopp M., Kloareg B., Fritig B. (2000). Linear beta-1,3 glucans are elicitors of defense responses in tobacco. Plant Physiol..

[34] Mercier L., Lafitte C., Borderies G., Briand X., Esquerré-Tugayé M.T., Fournier J. (2001). The algal polysaccharide carrageenans can act as an elicitor of plant defence. New Phytol..

[35] Millet Y.A., Danna C.H., Clay N.K., Songnuan W., Simon M.D., Werck-Reichhart D., Ausubel F.M. (2010). Innate immune responses activated in *Arabidopsis* roots by microbe-associated molecular patterns. Plant Cell.

[36] Cheng Z., Li J.-F., Niu Y., Zhang X.-C., Woody O.Z., Xiong Y., Djonovic S., Millet Y., Bush J., Mcconkey B.J. (2015). Pathogen-secreted proteases activate a novel plant immune pathway. Nature.

[37] Liu X., Williams C.E., Nemacheck J.A., Wang H., Subramanyam S., Zheng C., Chen M.-S. (2010). Reactive oxygen species are involved in plant defense against a gall midge. Plant Physiol..

[38] Vandenabeele S., Van Der Kelen K., Dat J., Gadjev I., Boonefaes T., Morsa S., Rottiers P., Slooten L., Van Montagu M., Zabeau M. (2003). A comprehensive analysis of hydrogen peroxide-induced gene expression in tobacco. Proc. Natl. Acad. Sci. USA.

[39] Stella Maris® on Wine Grapes Improves Yield and Quality Replicated Field Study. http://www.bartlett.ca/Bartlett/nmb/MSDSLabel.nsf/0/BCA5429A2B9E636285257F94006D6870/$file/STE.ORG.4.4.E.0116_winegrapes_1312.pdf.

[40] Danna C.H., Millet Y., Koller T., Han S.-W., Bent A.F., Ronald P.C., Ausubel F.M. (2011). The *Arabidopsis* flagellin receptor FLS2 mediates the perception of *Xanthomonas* Ax21 secreted peptides. Proc. Natl. Acad. Sci. USA.

[41] Scarpeci T.E., Zanor M.I., Mueller-Roeber B., Valle E.M. (2013). Overexpression of *AtWRKY30* enhances abiotic stress tolerance during early growth stages in *Arabidopsis thaliana*. Plant Mol. Biol..

[42] De Vleesschauwer D., Xu J., Höfte M. (2014). Making sense of hormone-mediated defense networking: From rice to *Arabidopsis*. Front. Plant Sci..

[43] Nafisi M., Goregaoker S., Botanga C.J., Glawischnig E., Olsen C.E., Halkier B.A., Glazebrook J. (2007). *Arabidopsis* cytochrome P450 monooxygenase 71A13 catalyzes the conversion of indole-3-acetaldoxime in camalexin synthesis. Plant Cell.

[44] Huffaker A., Ryan C.A. (2016). Endogenous peptide defense signals in *Arabidopsis* differentially amplify signaling for the innate immune response. Proc. Natl. Acad. Sci. USA.

[45] Malinovsky F.G., Fangel J.U., Willats W.G.T. (2014). The role of the cell wall in plant immunity. Front. Plant Sci..

