# Supplementary figures and images for: Seaweed Extract (Stella Maris®) Activates Innate Immune Responses in Arabidopsis thaliana and Protects Host against Bacterial Pathogens
Source: Mar Drugs. 2018 Jun 28;16(7):221. doi: 10.3390/md16070221 (PMC6071235; doi:10.3390/md16070221)

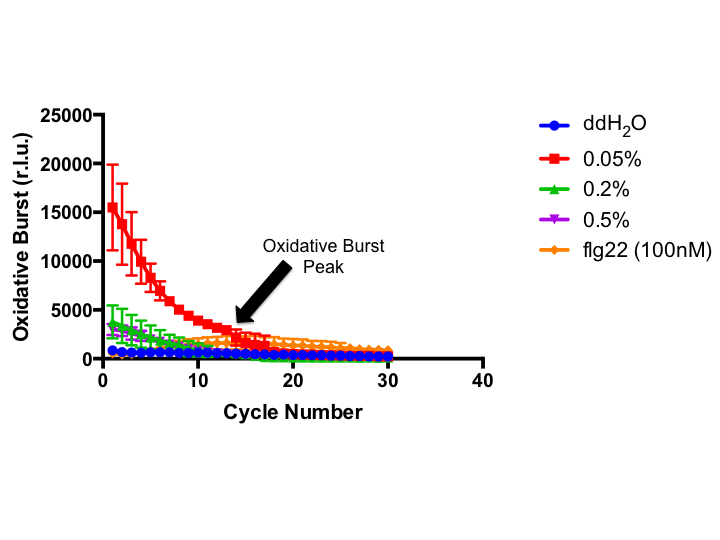

Supplement: Supplementary file 1 [file marinedrugs-16-00221-s001.zip › Marine Drugs Supplementary Figures/Supplementary Figure 1.tif]

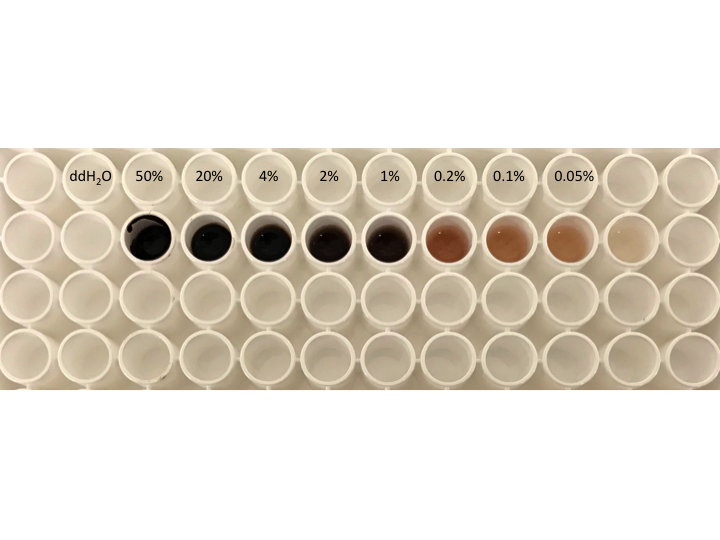

Supplement: Supplementary file 1 [file marinedrugs-16-00221-s001.zip › Marine Drugs Supplementary Figures/Supplementary Figure 2.tif]

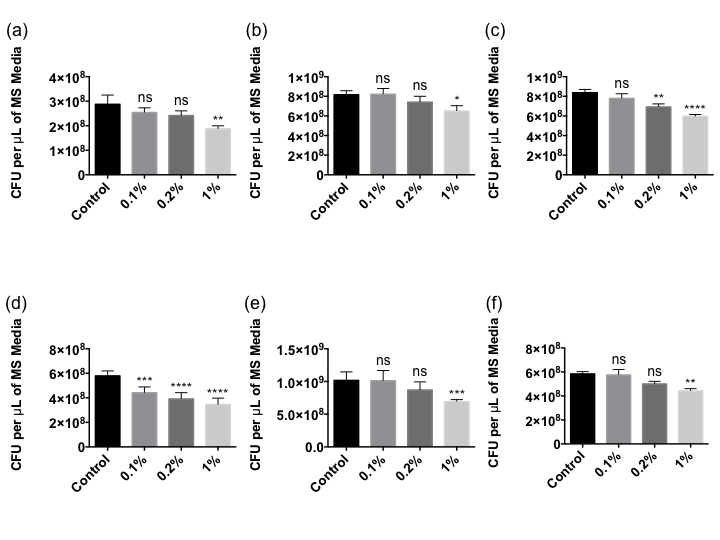

Supplement: Supplementary file 1 [file marinedrugs-16-00221-s001.zip › Marine Drugs Supplementary Figures/Supplementary Figure 3.tif]
